# Supplementary material for: Direct Measurement of the Four-Phase Equilibrium Coexistence Vapor–Aqueous Solution–Ice–Gas Hydrate in Water–Carbon Dioxide System
Source: Int J Mol Sci. 2023 May 26;24(11):9321. doi: 10.3390/ijms24119321 (PMC10253777; doi:10.3390/ijms24119321)
Supplement: Supplementary file 1 [file ijms-24-09321-s001.zip › ijms-2408302-supplementary.pdf]

Direct Measurement of the Four-Phase Equilibrium Coexistence Vapor–Aqueous Solution–Ice–Gas Hydrate in Water–Carbon Dioxide System

Anton Semenov [1,*], Rais Mendgaziev [1], Andrey Stoporev [1,2,*], Vladimir Istomin [1,3], Timur Tulegenov [1], Murtazali Yarakhmedov [1], Andrei Novikov [1] and Vladimir Vinokurov [1]

1 Department of Physical and Colloid Chemistry, Gubkin University, 65, Leninsky Prospekt, Building 1, 119991 Moscow, Russia; meda810@mail.ru (R.M.); vlistomin@yandex.ru (V.I.); tulegenov08@list.ru (T.T.); murtazali99@bk.ru (M.Y.); novikov.a@gubkin.ru (A.N.); vladimir@vinokurov.me (V.V.)
2 Department of Petroleum Engineering, Kazan Federal University, Kremlevskaya Str. 18, 420008 Kazan, Russia
3 Skolkovo Institute of Science and Technology (Skoltech), Nobelya Str. 3, 121205 Moscow, Russia
* Correspondence: semenov.a@gubkin.ru (A.S.); andrey.stoporev@kpfu.ru (A.S.)

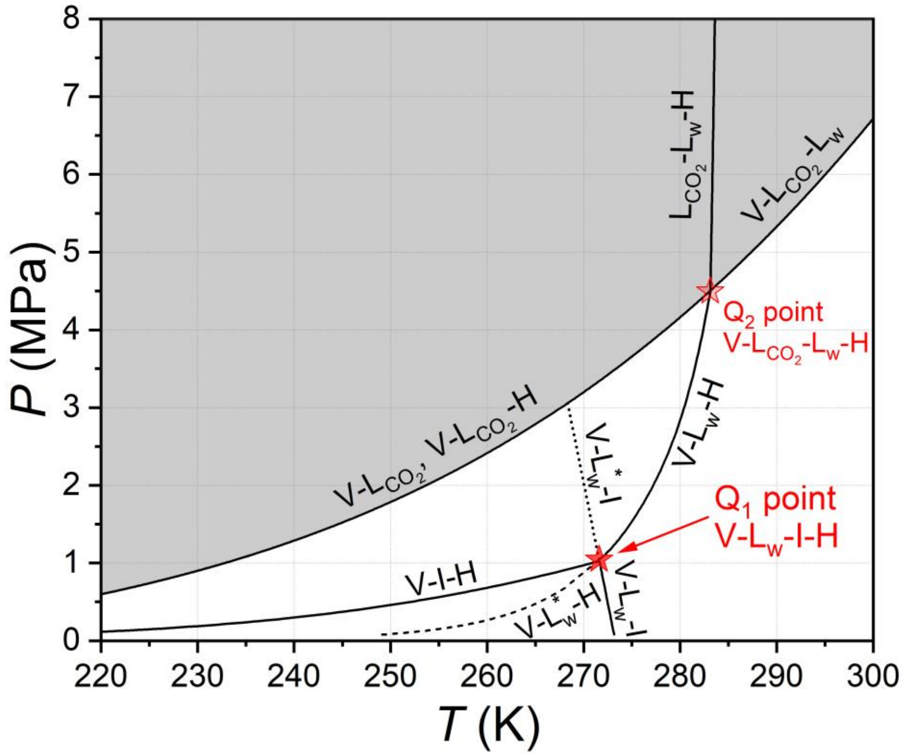

Figure S1. Phase diagram for the H_2O-CO_2 system based on literature data for the temperature and pressure range of 220–300 K and 0.08–8 MPa; gray and white colors correspond to the regions of existence of liquid and gaseous carbon dioxide, respectively; solid line V-L_w-H is the three-phase gas – aqueous solution – gas hydrate equilibrium (fit based on [1,2]), dashed line V-L_w^*-H is the metastable three-phase gas – supercooled aqueous solution – gas hydrate equilibrium (fit based on [3,4]), solid line V-I-H is the three-phase gas – ice – gas hydrate equilibrium (fit based on [5,6]), solid line V-L_w-I is the three-phase gas – liquid aqueous solution – ice equilibrium (fit based on [7]), dotted line V-L_w^*-I is the metastable three-phase gas – supercooled aqueous solution – ice equilibrium (fit based on [7]), solid line V-L_{CO_2} is the two-phase equilibrium of gaseous and liquid carbon dioxide (fit based on [8]) overlapping with solid line V-L_{CO_2}-(H or L_w) of the three-phase equilibrium of gaseous and liquid carbon dioxide and gas hydrate (or aqueous solution, fit based on [9,10]), solid line L_{CO_2}-L_w-H is the three-phase liquid carbon dioxide-rich phase – aqueous solution – gas hydrate equilibrium (fit based on [11,12]); the red stars represent the nonvariant four-phase equilibria: gas – aqueous solution – ice – gas hydrate (Q_1 point (271.60 K, 1.044 MPa), data of this work) and gas – liquid carbon dioxide-rich phase – aqueous solution – gas hydrate (Q_2 point, (283.13 K, 4.494 MPa), intersection of fits based on [1,2] and [9,10]).

Table S1. Numerical values of the coefficients of equation 1 for the H_2O-CO_2 system, describing pressure as a function of temperature of V-L_w-H equilibrium (based on our data [1,2])

Parameter	Value	Standard error
A	-2270.0193238	244.0937746
B	86815.7566903	10220.1927544
C	348.0192435	36.8410732
Adj. R-Square	0.99973	
AAD, MPa	0.016	
AARD, %	0.63	

Table S2. Numerical values of the coefficients of polynomial equation 2 for the H_2O-CO_2 system, describing pressure as a function of temperature of V-L_w-H equilibrium (based on our data [1,2])

Parameter	Value	Standard error
A_0	912403.6997181	15699.8646441
A_1	-13275.1064242	226.0452421
A_2	72.4389526	1.221061
A_3	-0.1757037	0.002933
A_4	$1.598395 \cdot 10^{-4}$	$2.643151 \cdot 10^{-6}$
Adj. R-Square	0.99997	
AAD, MPa	0.004	
AARD, %	0.19	

References

[1] A.P. Semenov, R.I. Mendgaziev, A.S. Stoporev, V.A. Istomin, D. V. Sergeeva, T.B. Tulegenov, V.A. Vinokurov, Dimethyl sulfoxide as a novel thermodynamic inhibitor of carbon dioxide hydrate formation, Chem. Eng. Sci. 255 (2022) 117670. https://doi.org/10.1016/j.ces.2022.117670.

[2] A.P. Semenov, R.I. Mendgaziev, A.S. Stoporev, V.A. Istomin, D. V. Sergeeva, T.B. Tulegenov, V.A. Vinokurov, Dataset for the dimethyl sulfoxide as a novel thermodynamic inhibitor of carbon dioxide hydrate formation, Data Br. 42 (2022) 108289. https://doi.org/10.1016/j.dib.2022.108289.

[3] Y. Nema, R. Ohmura, I. Senaha, K. Yasuda, Quadruple point determination in carbon dioxide hydrate forming system, Fluid Phase Equilib. 441 (2017) 49–53. https://doi.org/10.1016/j.fluid.2016.12.014.

[4] V.P. Melnikov, A.N. Nesterov, A.M. Reshetnikov, V.A. Istomin, Metastable states during dissociation of carbon dioxide hydrates below 273K, Chem. Eng. Sci. 66 (2011) 73–77. https://doi.org/10.1016/j.ces.2010.10.007.

[5] K. Yasuda, R. Ohmura, Phase Equilibrium for Clathrate Hydrates Formed with Methane, Ethane, Propane, or Carbon Dioxide at Temperatures below the Freezing Point of Water, J. Chem. Eng. Data. 53 (2008) 2182–2188. https://doi.org/10.1021/je800396v.

[6] H.D. Nagashima, N. Fukushima, R. Ohmura, Phase equilibrium condition measurements in carbon dioxide clathrate hydrate forming system from 199.1 K to 247.1 K, Fluid Phase Equilib. 413 (2016) 53–56. https://doi.org/10.1016/j.fluid.2015.09.020.

[7] V.P. Mel'nikov, A.N. Nesterov, L.S. Podenko, A.M. Reshetnikov, Influence of carbon dioxide on melting of underground ice, Dokl. Earth Sci. 459 (2014) 1353–1355. https://doi.org/10.1134/S1028334X14110245.

[8] W. Duschek, R. Kleinrahm, W. Wagner, Measurement and correlation of the (pressure, density, temperature) relation of carbon dioxide II. Saturated-liquid and saturated-vapour densities and the vapour pressure along the entire coexistence curve, J. Chem. Thermodyn. 22 (1990) 841–864. https://doi.org/10.1016/0021-9614(90)90173-N.

[9] S.D. Larson, Phase Studies of the Two-Component Carbon Dioxide-Water System, Involving the Carbon Dioxide Hydrate, University of Illinois, Urbana, IL, 1955.

[10] J.G. Vlahakis, H.S. Chen, M.S. Suwandi, A.J. Barduhn, The Growth Rate of Ice Crystals: Properties of Carbon dioxide Hydrates, A Review of Properties of 51 Gas Hydrates, 1972.

[11] S. Takenouchi, G.C. Kennedy, Dissociation Pressures of the Phase $CO_2 \cdot 5\ 3/4\ H_2O$, J. Geol. 73 (1965) 383–390. https://doi.org/10.1086/627068.

[12] K.I. Adeniyi, C.E. Deering, E. Grynia, R.A. Marriott, Water content and hydrate dissociation conditions for carbon dioxide rich fluid, Int. J. Greenh. Gas Control. 101 (2020) 103139. https://doi.org/10.1016/j.ijggc.2020.103139.
